# Supplementary figures and images for: Electrographic Seizures in Neonates with a High Risk of Encephalopathy
Source: Children (Basel). 2022 May 24;9(6):770. doi: 10.3390/children9060770 (PMC9221774; doi:10.3390/children9060770)

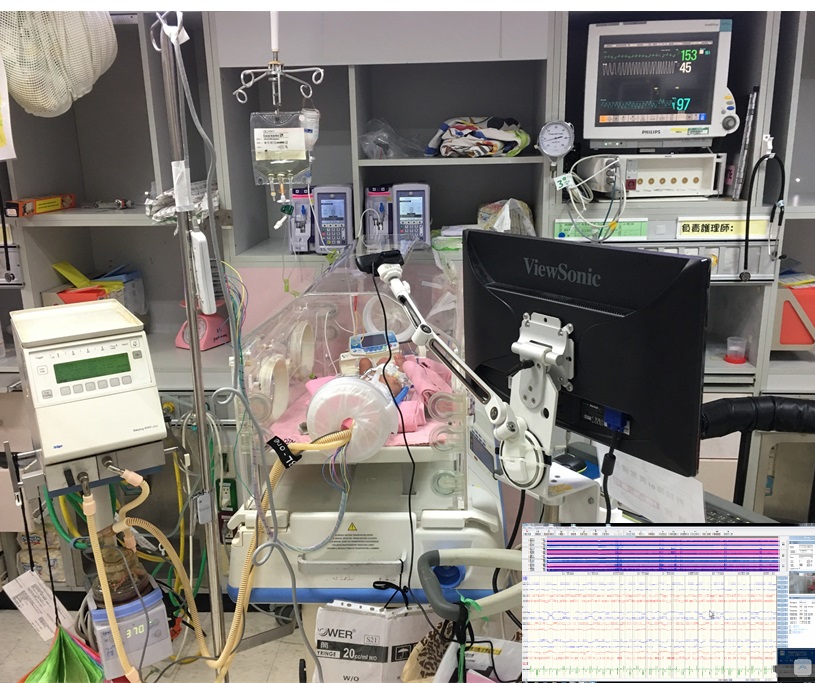

Supplement: Supplementary file 1 [file children-09-00770-s001.zip › 2022-03-26 Supplement Figure S1-cEEG monitoring system.jpg]

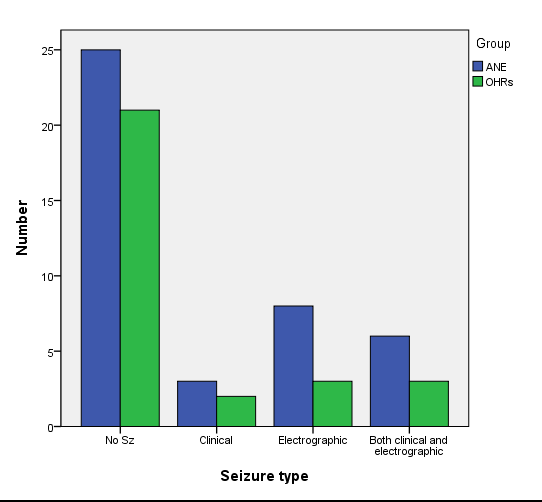

Supplement: Supplementary file 1 [file children-09-00770-s001.zip › 2022-03-26 Supplement Figure S2 Seizure type in 2 groups.tif]
